# Multidisciplinary approach for acute MI complicated by ventricular tachycardia, stent thrombosis, and multi‐organ failure: A case report

**DOI:** 10.1002/ccr3.8442

**Published:** 2024-01-29

**Authors:** Ujjwal P. Dutta, Jugal Hiren Bhatt, Irfan Nagori, Nency Kagathara, Rushit Zalavadiya, Dilip Sapkota

**Affiliations:** ^1^ Department of Internal Medicine GMERS Medical College Gotri Vadodara Gujarat India; ^2^ Department of Internal Medicine Zydus Medical College and Hospital Dahod India; ^3^ Department of Medicine Bharatpur Hospital Chitwan Nepal

**Keywords:** hypoxic brain injury, myocardial infarction, stent thrombosis, ventricular tachycardia

## Abstract

**Key Clinical Message:**

The case highlights the imperative requirement for multidisciplinary action in handling a myocardial infarction case, complicated by rare and severe events like ventricular tachycardia, stent thrombosis, hypoxic brain injury, and multi‐organ failure.

**Abstract:**

This article presents a case of a 53‐year‐old male, who presented with myocardial infarction that was managed by percutaneous coronary intervention and stent placement. However, it progressed to multiple complications in sequence (ventricular tachycardia, stent thrombosis, hypoxic brain injury, and multi‐organ failure). Hopefully, the condition of the patient improved after 2 months from GSC‐4 to GCS‐9 by a multidisciplinary approach and was discharged for home‐based treatment.

## 1 INTRODUCTION

Acute myocardial infarction (MI) requires urgent intervention to restore coronary blood flow and prevent adverse outcomes.^1^ Percutaneous coronary intervention (PCI) with stent placement is the standard for revascularization, but complications like stent thrombosis and ventricular arrhythmias can lead to significant morbidity and mortality.^2^, ^3^

This case involves a patient with acute MI experiencing complex complications, including ventricular tachycardia, stent thrombosis, hypoxic brain injury, pulmonary edema, and multi‐organ failure. A multidisciplinary approach is crucial for optimal patient outcomes, with early recognition, prompt intervention, and meticulous critical care management being vital in high‐risk cases.^4^

Effective management involves collaboration between cardiologists, intensivists, neurologists, and critical care specialists.^5^ Utilizing evidence‐based guidelines and advanced medical therapies, such as mechanical circulatory support, contributes to improved survival rates.^6^ Ongoing research and innovation in critical care are essential for enhancing patient outcomes.^7^

## 2 CASE PRESENTATION

**FIGURE 1:**
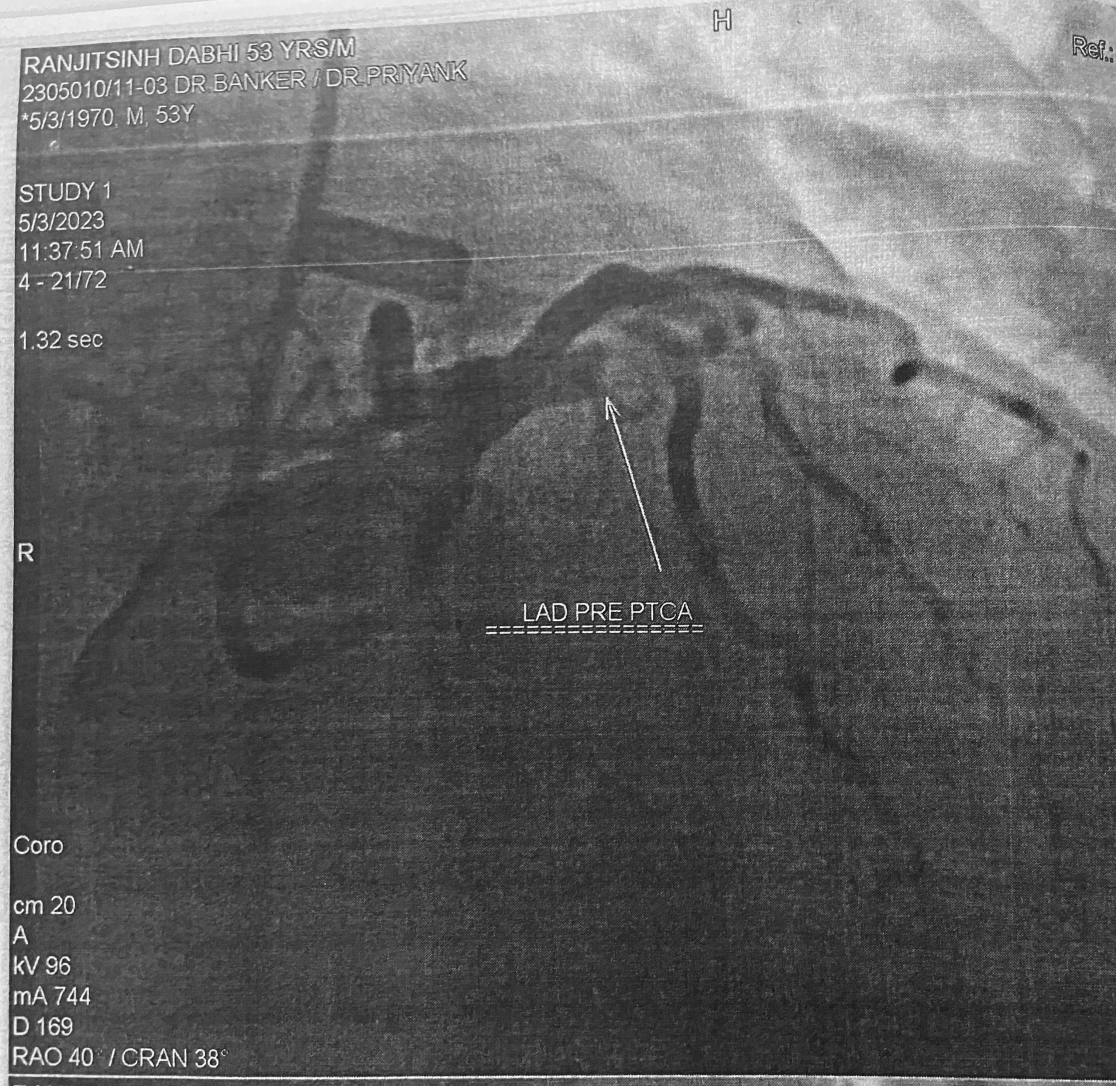
Coronary angiography of left anterior descending artery showing blockage (Pre‐PTCA).

A 53‐year‐old male with a history of hypertension and dyslipidemia was admitted to Tier‐1 private hospital's emergency department on 3 May 2023, unconscious and experiencing sudden‐onset severe chest pain radiating to the left arm. Urgent resuscitation efforts, including CPR and DC shock, restored normal sinus rhythm, and the patient was intubated. ECG indicated ST‐segment elevation in multiple leads, suggesting an occlusion in the left anterior descending artery (LAD). Urgent coronary angiography (Figure 1) confirmed a 95% LAD occlusion with no collaterals visible, diagnosing an anterior wall myocardial infarction (AWMI).

**FIGURE 2:**
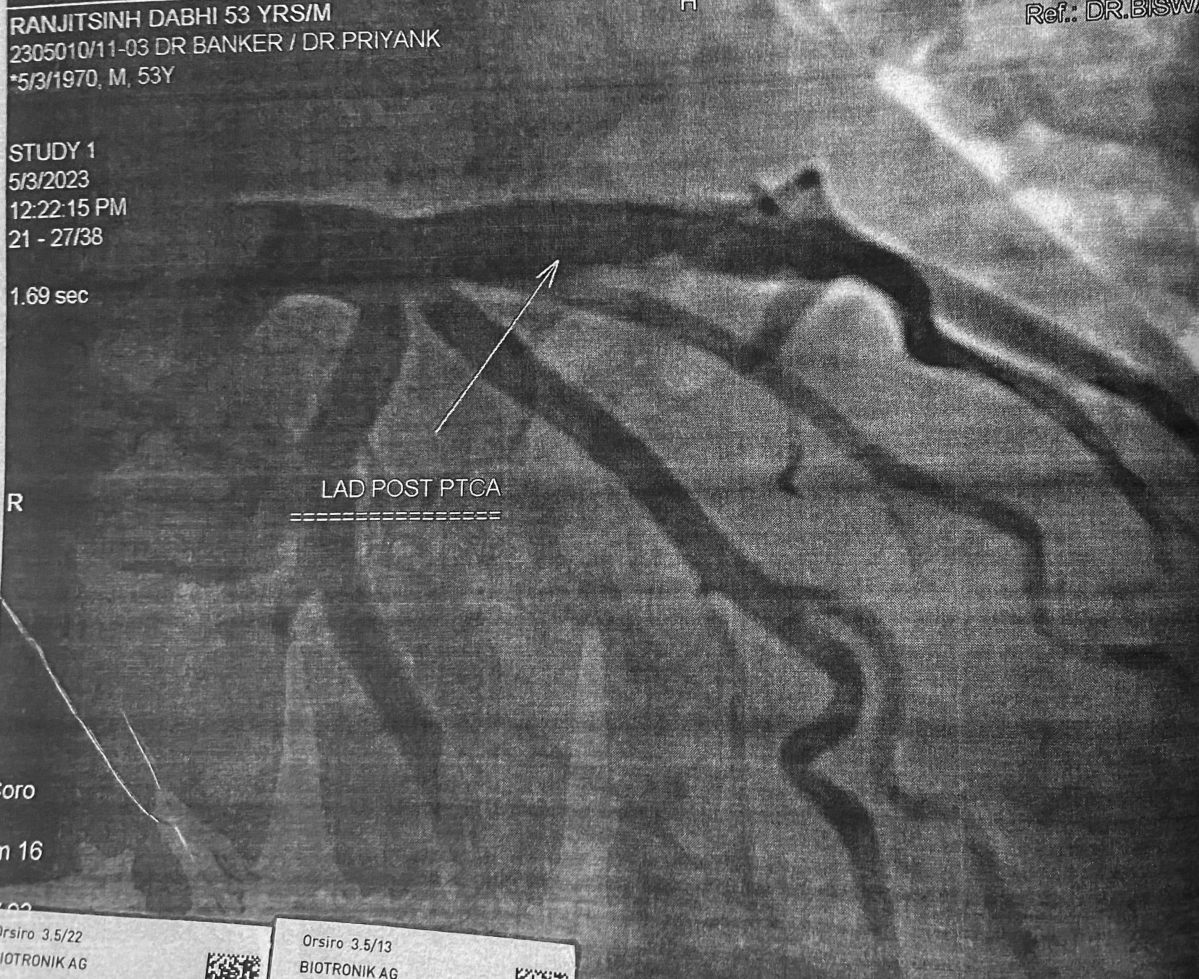
Coronary angiography of left anterior descending artery after pci and stent placement (Post‐PTCA).

**FIGURE 3:**
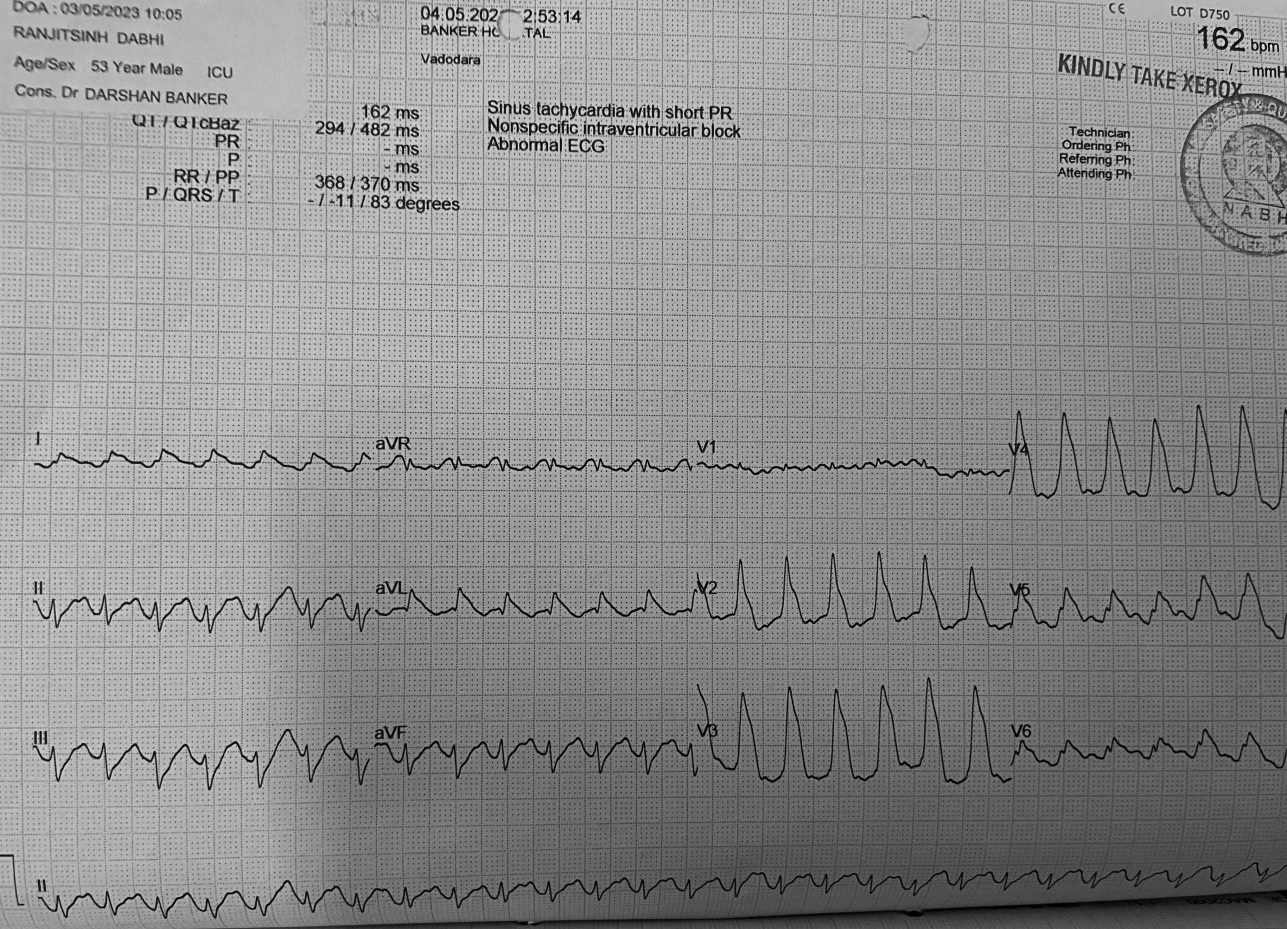
ECG depicting ventricular tachycardia.

Immediate percutaneous coronary intervention (PCI) with a drug‐eluting stent (Figure 2) in the LAD was performed. Despite successful initial management, the patient developed ventricular tachycardia (Figure 3) on the same day, requiring DC shock and reintubation. Coronary angiography revealed stent thrombosis, leading to reduced cardiac index and systemic perfusion. Despite resuscitation efforts, surgical thrombolysis and stent replacement, the patient's condition worsened, resulting in hypoxic brain injury, necessitating ventilatory support.

**FIGURE 4:**
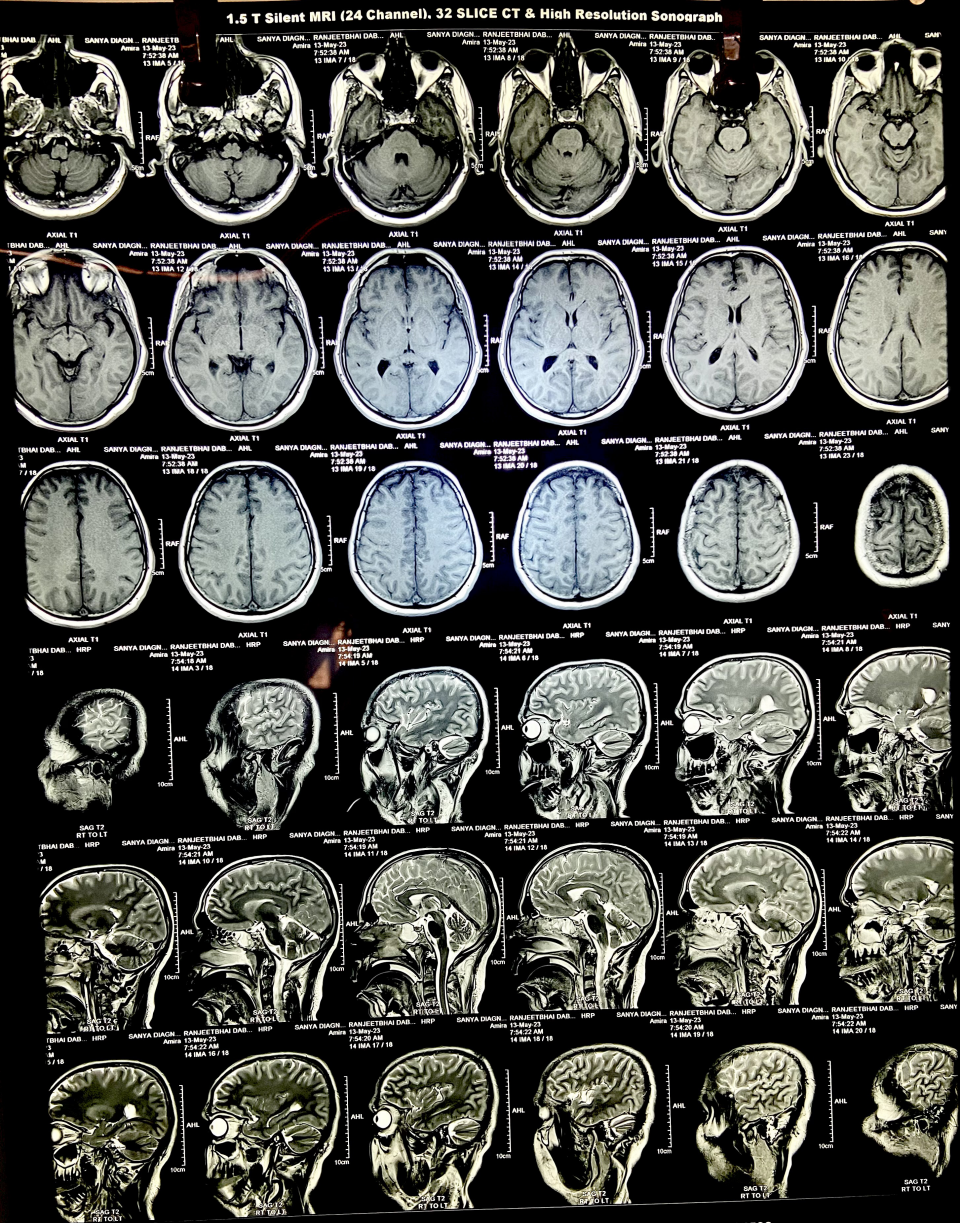
An MRI of the brain, depicting multifocal hypoxic brain injury.

**FIGURE 5:**
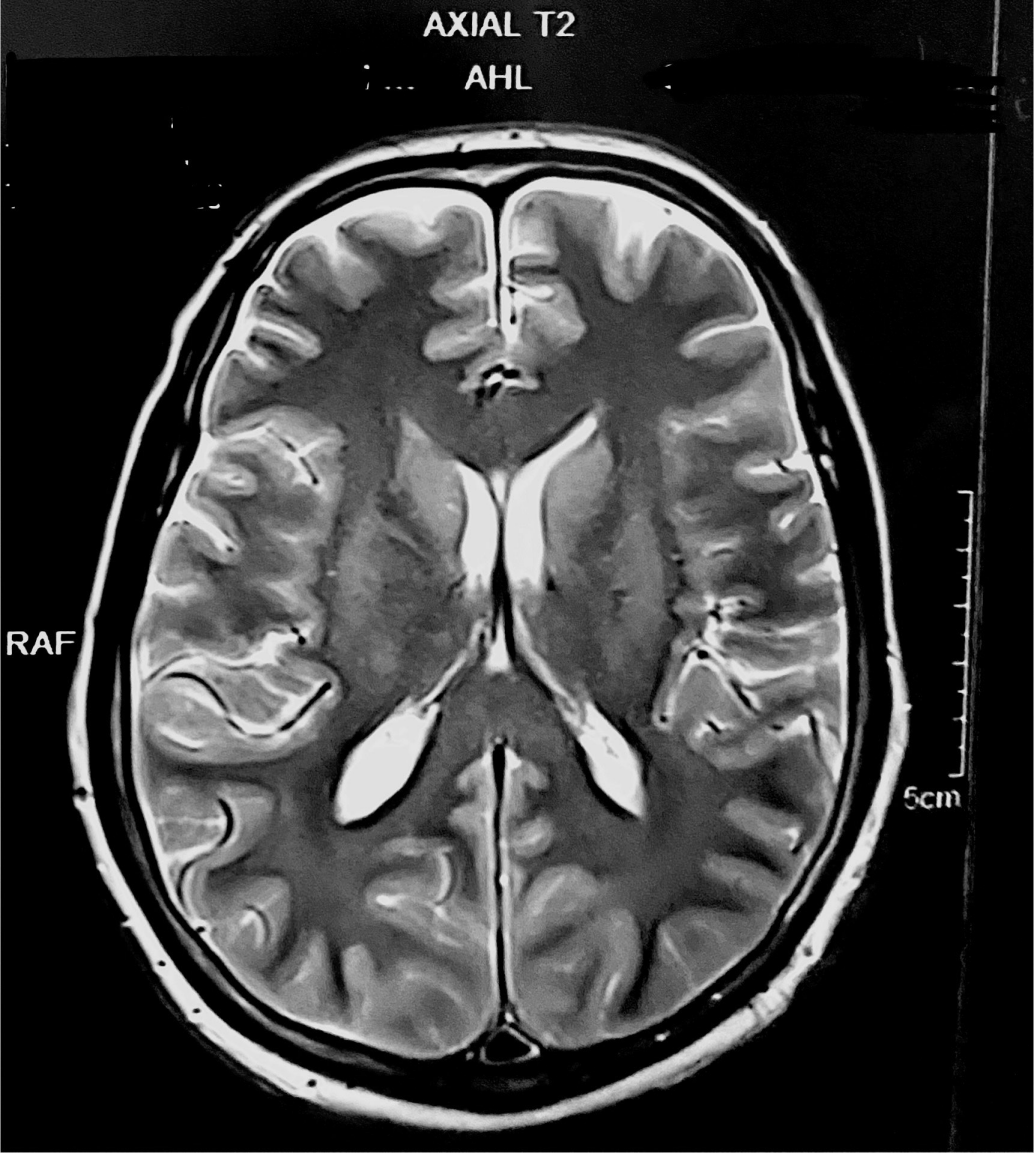
Axial T1 view of MRI brain.

**FIGURE 6:**
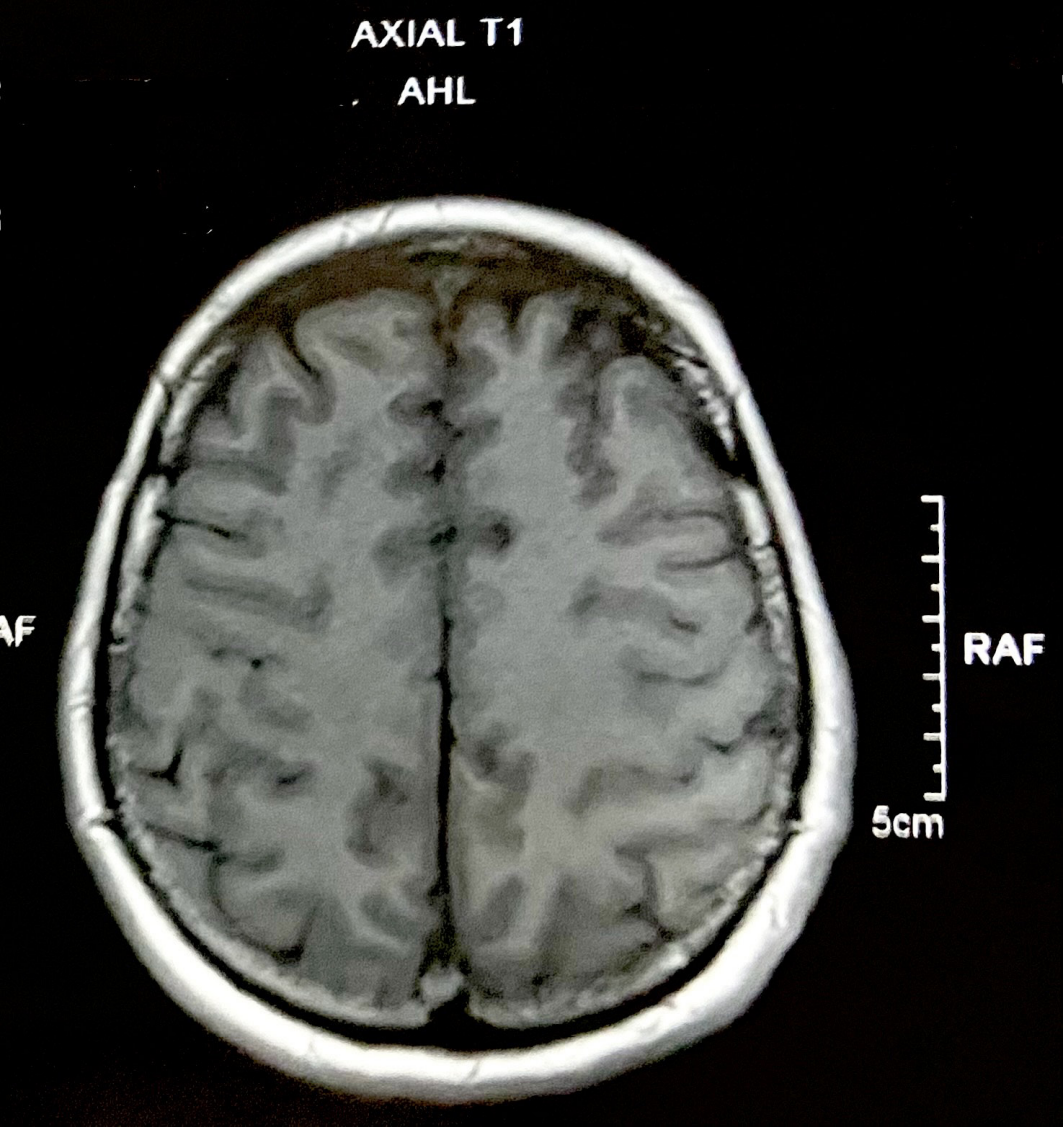
Axial T2 view of MRI brain.

An MRI of the brain (Figures 4, 5, 6) at a local private hospital on 8th May 2023 revealed evidence of diffuse restriction involving bilateral fronto‐parietal white matter including centrum semiovale, corona radiata and corpus callosum. A similar involvement was also seen in the posterior limb of internal capsule and globus pallidus bilaterally. Also, vasogenic edema was observed involving bilateral fronto‐parieto occipital and bilateral basal ganglia significantly suggestive of hypoxic brain injury involving the cerebrum.

The patient remained vitally stable maintaining a SpO2 between 95% and 99%, heart rate fluctuated between 78 bpm to 119 bpm and blood pressure varying from but remaining within the range of 112–126 systolic and 76–82 diastolic with strict 2 hourly monitoring of vitals including temperature and random blood glucose levels both of which remained within the normal physiological limits.

Due to the poor economic condition of the patient and his financial capabilities along with the rising expenditure for the ICU stay in the private hospital the patient was subsequently admitted to a government hospital's medical intensive care unit (MICU) in an unconscious state on 16 May 2023.

In the MICU, the patient gradually developed pulmonary edema, requiring ventilatory support with positive end‐expiratory pressure (PEEP) for optimal oxygenation. Adequate maintenance of SpO2 levels between 97% and 99% was achieved, vitals including blood pressure, pulse rate, temperature, fluid input/output charting and random blood glucose levels were monitored every 2 h but on the sixth day, a sudden drop in SpO2 levels to 40% required immediate intervention.

Following FiO_2_ increase to 100%, the patient's SpO_2_ very gradually improved to 94%–96% and after a few hours reached 98%–100%. The patient had intact ciliary and pupillary reflexes, lacked deep and superficial tendon reflexes, with spontaneous eye opening. With a GCS score of 4 at admission, the patient remained in a quadriplegic and comatose state in the MICU for 59 days until July 14, 2023 followed by a 7 day stay at the inpatient medicine wards.

During this stay, the ventilator settings were set to CPAP mode with PEEP of 5 mm of Hg, FiO_2_ of 70%, which was adequate to maintain SpO2 levels at a relatively constant value between 98% and 100%. The patient was responding to deep stimuli and there was spontaneous eye opening. The tracheostomy tube was properly and thoroughly cleaned by suctioning combined with administration of 2 mL of sodium bicarbonate solution intratracheally by the MICU physician to loosen up and prevent formation of any secretion building and ease the suctioning process to ensure a clean and fully patent tracheostomy tube for adequate air flow in and out. The Department of Otorhinolaryngology was consulted weekly once to manage the tracheostomy tube placed by various means such as regular changing of the dressing around the tube, daily morning suctioning with sodium bicarbonate and regular checks of the patency and position of the tube to ensure adequate ventilation of the patient. To prevent sucreation/mucus build up and loosening up of the existing secretions regular nebulisation therapy was given with 1 mL of ipravent (ipratropium) solution and 1 2 mL Budecort Respule containing 0.5 mg of budesonide. All these measures ensured prevention of tracheostomy tube blockage and maintenance of a completely patent tube for proper ventilation.

With respect to medication, the patient was started on injectable medications including a series of antibiotics and other routine medications. One pint (500 mL) of normal saline was administered once daily along with a multivitamin injection (MVI) + B12 that was also given intravenously in 100 mL normal saline separately. During the first few days, the patient developed moderate peripheral and facial edema, which was managed with intravenous administration of Lasix (Furosemide) 40 mg BD while maintaining his physiological blood pressure. Injection of Levipil (Levetiracetam) 500 mg BD was also administered for the first 7 days. Regarding antibiotics, ceftriaxone 1 gm iv was given every 12 h along with an injection of colistin (Colistimethate Sodium—two million units iv, every 12 h) for the first 14 days.

After the first week, the patient developed some trophic ulcers (pressure ulcers) in the buttock region and on the posterior aspect of the right heel. A surgical consult was conducted in order to manage and prevent these ulcers. It was managed with frequent changes in position of the patient and with an addition of a 21 day course of injectable antibiotics such as meropenem 1 gm iv every 8 h, clindamycin 600 mg every 12 h, linezolid 600 mg every 12 h, to prevent any bacterial growth and infection of the ulcerated sites and development of septicemia and shock. The prevention of these was ensured by a weekly consult of the surgical department to detect and manage any other ulcers if present while maintaining a strict control and monitoring over his blood glucose levels to avoid hyperglycemia. Other routine IV injectables were pantoprazole 40 mg and emeset (Ondansetron) 4 mg every 12 h. Some additional routine medications were given as tablets crushed into powder form mixed with clean drinking water at room temperature through the Ryle's tube (Nasogastric tube) such as aspirin 150 mg and clopidogrel 75 mg to prevent hypercoagulability, clot formation, as a preventive measure for further thrombotic episodes. Similarly atorvastatin 40 mg was also given once daily to prevent hyperlipidemia and prevent any formation and/or progression of atherosclerotic activity in the circulation. Multivitamin tablets and folic acid tablets were given every 12 h along with calcium tablets given once daily. To prevent constipation Syrup laxose (Lactulose) 2 Tbsp was given three times daily along with regular PC enemas.

A daily consultation with the Department of Physiotherapy was also arranged from 7th day onwards to ensure sufficient movement and exercise to prevent severe disuse atrophy of the muscles and formation of pressure areas over the back, heels, and buttocks.

From the 5th of July it was decided to begin spontaneous room air trials of 5–10 min each to assess the respiratory drive of the patient and his capability to wean off the ventilator but unfortunately his Spo2 levels kept on dropping to below 85% after some time on room air followed by immediate reverting to the ventilatory support and improvement of Spo_2_ levels to the original values. The GCS level was E3VtM1. Room air trials were continued and a gradual improvement was seen in the patient by a gradual cessation of drops in Spo2 levels.

On 8 July 2023, the patient maintained a relatively stable Spo2 levels of 94%–97% on three consecutive room air trials. Followed by this, a joint decision was taken by the Departments of Medicine and Otorhinolaryngology to initiate stripping of the tracheostomy tube and gradually shifting the patient to nasal prongs ventilation at 3 L/minute of O_2_ flow. This was done gradually and the Spo2 levels were periodically monitored to check for any sudden drops in oxygen levels.

On 11 July 2023, upon confirmation of the patient's healthy respiratory drive and capacity to maintain blood oxygen levels within the physiological parameters, it was decided to remove the tracheostomy tube and to shift the patient to nasal prongs for ventilation entirely. Following this, the patient was shifted to the medicine male ward on 14 July 2023 for further management and rehabilitation.

After discharge from the MICU, the patient's GCS score had improved to 9, indicating significant neurological progress. The routine medications both injectables and oral were continued in the inpatient wards with the addition of azithromycin 500 mg and an injection of cefo sulbactam 1.5 gm once a day for 7 days. Intensive physiotherapy addressed the limb mobility issues, but complete restoration and effective communication remained impaired. Surgical consultations were sought for managing trophic ulcers on the buttocks and both heels, emphasizing interdisciplinary care from neurology, physiotherapy and wound specialists.

Throughout therapy, the patient exhibited improved arousal and neurological responsiveness, although further progress was needed for limb mobility and communication. Home‐based treatment aimed to enhance recovery and foster independence, with frequent follow‐up evaluations to monitor progress after the discharge of the patient on 20 July 2023.

**FIGURE 7:**
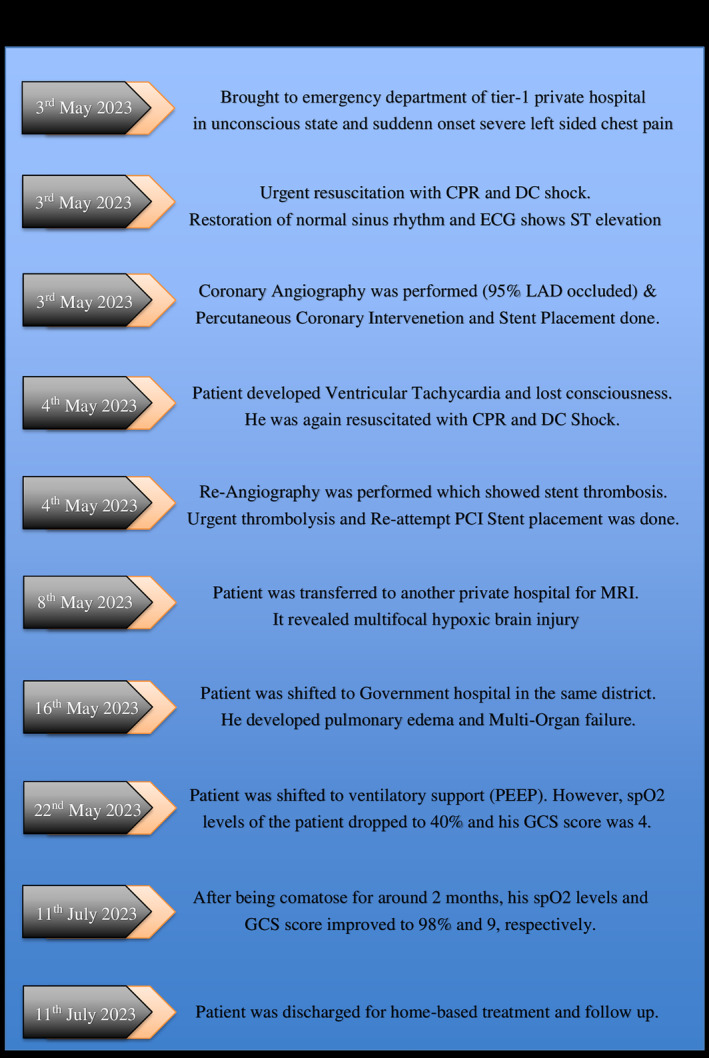
Timeline of the complete case scenario of the patient.

This complex clinical scenario (Figure 7) highlights the need for a multidisciplinary approach and advances in critical care management to optimize patient outcomes in severe multi‐organ illnesses.

## 3 DISCUSSION

The presented case underscores the intricate challenges faced in the management of severe case of myocardial infarction with multiple severe rare complications. His sudden presentation at the Tier‐1 private hospital's emergency department with unconsciousness, severe chest pain, and subsequent diagnosis of anterior wall myocardial infarction (AWMI) reflects the critical nature of the situation.^1^ The most common risk factor associated with STEMI is hypertension and the most implicated factors in AWMI that lead to hemodynamic compromise are a history of Diabetes Mellitus, severe left ventricular (LV) systolic dysfunction, elevated troponin‐T levels, and no collaterals that correlate with worse outcomes. Our patient had a long‐term history of hypertension and dyslipidemia, no collaterals formed, and severe LV systolic dysfunction, which jointly led to worse outcomes of AWMI, that is hypoxic brain injury followed by coma.^8^, ^9^

The swift response of the medical team, encompassing urgent resuscitation efforts, percutaneous coronary intervention (PCI), and subsequent interventions, illustrates the comprehensive and dynamic nature of critical care management. However, the patient's clinical course was marked by unforeseen complications, including ventricular tachycardia, followed by stent thrombosis and subsequent hypoxic brain injury. The incidence rate of ventricular tachycardia and stent thrombosis after PCI and stent placement is 3.2–5.7% and <1% respectively.^10^, ^11^ The incidence of both these severe and rare complications through the course of condition at the same time made it unique and out of the common, highlighting the unpredictable and complex nature of medical conditions.

Following these events, thrombolysis and re‐placement of stent was done immediately after diagnosing stent thrombosis on coronary angiography, as it is crucial for prevention of further ischemic damage to cardiac wall, if not performed it might result in acute left ventricular failure. However, it is worth noting that re‐attempt of stent placement has lesser success rates as compared to the first stent placement.^12^ Notably, in this case there were no such stent related complications or restenosis after PCI‐stent re‐attempt and patient was stabilized which clearly highlights the importance of performing crucial intervention accurately at the peak of dreadful events.

The journey from immediate resuscitation to the subsequent development of neurological deficits necessitated a multidisciplinary approach, combining the expertise of cardiology, neurology, intensive care specialists, physiotherapists, and wound care specialists. According to a study, patients with GCS <6 had only 41.7% survival rate and another study found that patients with GCS between 3 and 5 had very high mortality.^13^, ^14^ In our case, the progression from an unconscious, quadriplegic, and comatose state with GCS score 4 to eventual neurological improvements with GCS score 9 further exemplifies the resilience of both the patient and the interdisciplinary care team. The transition from the medical intensive care unit (MICU) to home‐based care and improvement in spO_2_ levels from 40% to 98% further highlights the importance of sustained efforts to optimize recovery and foster independence. This complex case serves as a testament to the advancements in critical care management, as well as the imperative for ongoing research and collaboration among medical specialties to refine strategies for improving outcomes in severe multi‐organ illnesses.

## 4 CONCLUSION

This case report emphasizes the complexities encountered in managing a patient with acute MI complicated by ventricular tachycardia, stent thrombosis, hypoxic brain injury, pulmonary edema, and multi‐organ failure. The case highlights the need for early recognition, prompt intervention, and comprehensive critical care management in order to improve outcomes in such complex cases. Further research and advancements in critical care management strategies are warranted to optimize the prognosis for patients with severe multi‐organ illnesses.

## AUTHOR CONTRIBUTIONS

**Ujjwal P. Dutta:** Conceptualization; data curation; writing – original draft; writing – review and editing. **Jugal Hiren Bhatt:** Conceptualization; data curation; writing – original draft; writing – review and editing. **Irfan Nagori:** Conceptualization; supervision; writing – review and editing. **Nency Kagathara:** Conceptualization; writing – original draft. **Rushit Zalavadiya:** Conceptualization; data curation; writing – original draft. **Dilip Sapkota:** Conceptualization; supervision; writing – review and editing.

## FUNDING INFORMATION

This article received no funding.

## CONFLICT OF INTEREST STATEMENT

There are no conflicts of interest.

## CONSENT

Written informed consent was obtained from the patient for the publication of this case report and accompanying images, in accordance with the journal's patient consent policy. A copy of written consent is available for review by the Editor‐in‐Chief of this journal on request.

## Data Availability

The data that support the findings of this study are available on request from the corresponding author.
